# Sleep Disorders and Their Associated Factors during the COVID-19 Pandemic: Data from Peruvian Medical Students

**DOI:** 10.3390/medicina58101325

**Published:** 2022-09-22

**Authors:** Cesar Copaja-Corzo, Brayan Miranda-Chavez, Dariela Vizcarra-Jiménez, Miguel Hueda-Zavaleta, Marco Rivarola-Hidalgo, Edgar G. Parihuana-Travezaño, Alvaro Taype-Rondan

**Affiliations:** 1 Universidad Privada de Tacna, Tacna 23003, Peru; 2 Red Asistencial Ucayali EsSalud, Pucallpa 25003, Peru; 3 Hospital III Daniel Alcides Carrión EsSalud, Tacna 23000, Peru; 4 Unidad de Investigación para la Generación y Síntesis de Evidencias en Salud, Universidad San Ignacio de Loyola, Lima 15024, Peru; 5 EviSalud-Evidencias en Salud, Lima 15001, Peru

**Keywords:** Pittsburgh Sleep Quality Index, Peru, nomophobia, sleep quality, students, medical

## Abstract

*Background and objectives:* Sleep disorders are a common public health problem among college students. The objective of this study was to evaluate sleep quality and its associated factors in medical students during the COVID-19 pandemic. *Materials and Methods:* Cross-sectional analytical study—we conducted a secondary analysis of the survey “Nomophobia in medical students in Peru” database between 2020 and 2021. Sleep disturbances were assessed using the Pittsburgh Sleep Quality Index (PSQI). To evaluate associated factors, crude and adjusted prevalence ratios (aPR) and their 95% confidence intervals (95% CI) were calculated. *Results:* We analyzed data from 3139 participants from 18 cities in Peru (61.1% were women, median age: 22 years). 43.4% had a quality of sleep that could require medical attention; the PSQI dimension with the highest score was daytime dysfunction. The poor sleep quality was associated with symptoms of anxiety (aPR: 1.48; 95% CI: 1.27–1.72), depression (aPR: 2.03; 1.72–2.39), or nomophobia (aPR: 1.28; 1.09–1.51). *Conclusions:* Sleep disorders were a common problem among Peruvian medical students and were associated with anxiety, depression, or nomophobia symptoms.

## 1. Introduction

The COVID-19 pandemic has forced countries worldwide to implement measures to contain its spread, such as physical distancing, using masks, and, ultimately, mandatory social confinement [1]. In Peru, a state of emergency was decreed on 15 March 2020, and the beginning of compulsory social immobilization, which over time became focused on some regions during specific hours of the day and ended ultimately only on 31 January 2022 [2]. These measures, coupled with increased morbidity and mortality from COVID-19, have caused psychosocial problems such as stress, worry, fear, anxiety, depressive symptoms, and sleep disorders in public [3,4,5,6].

Sleep, like food, water, and air, is a physiological requirement for life. Common sleep disorders include insufficient sleep duration or poor daytime functioning [7]. These sleep disturbances were a public health problem long before the pandemic started [8]. Still, recent research reports that these problems could be increasing due to multiple factors caused by COVID-19, especially in specific populations such as university students, since most do not consider sleep a priority [9,10,11]. Among them, medical students are particularly prone to unhealthy lifestyles due to high academic load and poor time management skills, which make them more susceptible to sleep disorders and their consequences [12,13].

A systematic review that included 57 observational studies reported that the prevalence of poor sleep quality in medical students was 52.7%, with a variation between 45.3% and 60.1%, with an increase, especially in students from Europe and America [14], but this review only included studies that had been carried out before the COVID-19 pandemic and only included seven studies carried out in Latin America. In addition, the measures to contain the pandemic and its impact on the population were very diverse in each country. Therefore, it is essential to learn more about this topic in various contexts, especially in South American countries. In this sense, the objective of the following study was to evaluate the quality of sleep in medical students in Peru and its associated factors.

## 2. Materials and Methods

### 2.1. Study Design

Cross-sectional analytical study. We performed a secondary analysis of the survey “Nomophobia and its Associated Factors in Peruvian Medical Students.” This study aimed to evaluate the factors associated with nomophobia in Peruvian medical students. The methods used and the main results have been previously described [15].

### 2.2. Participants and Procedures

The primary study’s population included adult medical students (over 18 years of age) who agreed to participate and declared that they were enrolled in a human medicine school in Peru. Students who did not own a cell phone in the month before the survey were excluded.

The preliminary study performed a convenience sampling and collected information through an online survey using the Google form, following the described methodology. First, they disseminated the survey through social networks with a selective focus on medical students and provide incentives for completing the study altogether. Then they invited more researchers from different medical schools in Peru, and after the training, they were asked to make personal invitations to each medical student from their respective school.

### 2.3. Variable Definition

The present study focused on sleep quality as the primary outcome, which was assessed using the Pittsburgh Sleep Quality Index (PSQI) [16]. The PSQI comprises 24 items, of which 19 are self-applicable (with scores from 0 to 3 for each item), and five must be answered by a roommate. This last section is not essential to evaluate the PSQI. The global rating is obtained from the sum of the components where a higher score is indicative of a lower quality of sleep.

This way, the global classification can score between 0 and 21. The interpretation, according to the PSQI score obtained is presented as: no sleep problems (<5 points); deserves medical attention (5 to 7 points); deserves attention and treatment doctor (8 to 14 points); it is a severe sleep problem (≥15 points) [16]. For this study, we used 7 points as the cut-off point, those who had a score less than or equal to 7 were considered to have good sleep quality, and those who had a higher score were considered to have poor sleep quality. The study used the version validated in Spanish in medical students from Spain, which reported optimal internal consistency (Cronbach’s α coefficient of 0.81) [17].

To assess the degree of anxiety and depression, they used the Hopkins Symptom Checklist-25 (HSCL-25) [18]. The HSCL-25 consists of 25 items (10 to address anxiety and 15 to address depression). Responses are scored on a 4-point Likert scale, ranging from 1 (“not at all”) to 4 (“a lot”). The study used the version validated in Spanish in the adult Peruvian population, presenting adequate internal consistency (global Cronbach’s Alpha 0.90; anxiety, α = 0.81; and depression, α = 0.86) [19].

The severity of nomophobia was assessed using the Nomophobia Questionnaire (NMP-Q) [20]. The questionnaire consists of 20 items with a Likert scale score of 7 points ranging from 1 (“strongly disagree”) to 7 (“strongly agree”), and a total score between 20 and 140 points. For the study, they used the Spanish version of the NMP-Q, which was validated, reporting adequate internal consistency (Cronbach’s alpha 0.95) [21].

Other variables used for the analysis were: age (in terciles), sex, being in a relationship (yes/no), university financing (public/private), year of study (first to third year/fourth to the sixth year), failing a course in the previous semester (yes/no), number of siblings (none/one to two/more than 3), playing a sport (yes/no), level of higher technical or university education of the parents (yes/no), cell phone review time interval (5 min/20 min/1 h/3 h), cell phone usage schedule (all the time/morning/afternoon/night).

### 2.4. Statistical Analysis

We downloaded the database to the Microsoft Excel program and then exported it for analysis in the statistical program Stata v16. For the descriptive analysis, frequencies, percentages, measures of central tendency, and measures of dispersion were used.

To evaluate the factors associated with sleep quality, the dichotomous interpretation (good and poor sleep quality) of the PSQI was considered, as a result variable. We used Poisson regression models with robust variance and calculated the prevalence ratio (PR) with their respective 95% confidence intervals (95%CI). For this, we first performed a raw, robust variance Poisson regression with each variable, those with statistical significance (*p* < 0.05) were selected and finally entered the adjusted regression model.

### 2.5. Ethics

This study was conducted following the international research ethics guidelines of the Declaration of Helsinki. The research protocol was evaluated and approved by the ethics committee of the Faculty of Health Sciences of the Private University of Tacna (identification code: 099-FACSA-UI). Informed consent was not requested due to the observational and retrospective nature of the study.

## 3. Results

### 3.1. Population Characteristics

We analyzed data from 3139 medical students distributed in 18 cities in Peru. The female sex was predominant (61.1%), the median age was 22 years (IQR 20–24), and the majority reported that they did not play any sport (52.9%).

Regarding their education, 50.8% report that they study at a university with public financing, and 51.3% are between the 1st and 3rd year of studies. On the other hand, most students (86.5%) reported that they did not fail any course in the semester before the study.

Anxiety and depression symptoms were positive (score ≥ 1.75) in 34.8% and 42.8% of the students surveyed, respectively; likewise, the median nomophobia severity score was 47 points (IQR 33–69). On the other hand, most students had a quality of sleep that required medical attention (43.4%) (Table 1).

### 3.2. Distribution of Sleep Quality Score

**Figure 1:**
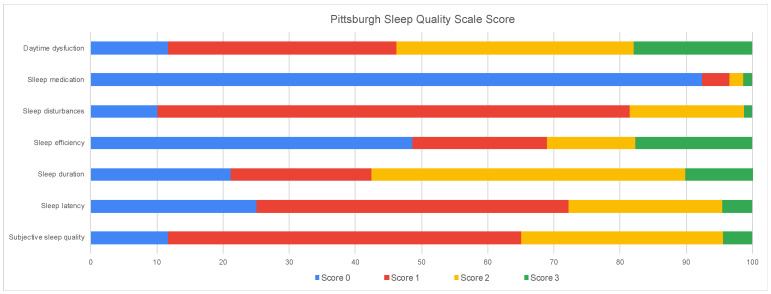
Distribution of the score of each dimension of the PSQI (*n* = 3139).

The dimension with the highest score (3 points) was daytime dysfunction, and within this, the highest item was “How much trouble have you had staying upbeat or enthusiastic when carrying out your tasks or activities?” (1 point) The second dimension with the highest score was sleep efficiency. On the other hand, the dimension with the lowest score was use of sleep medications, where most students report that they do not use sleep medications (Table 2 and Figure 1).

### 3.3. Factors Associated with Sleep Quality

In the adjusted Poisson regression model, we identified that the factors associated with poor sleep quality were anxiety symptoms (PRa: 1.48; 95% CI: 1.27–1.72), depression (PRa: 2.03; 95% CI: 1.72–2.39), or nomophobia (PRa: 1.28; 95% CI: 1.09–1.51). On the other hand, the factor associated with better sleep quality was found in those who reported using cell phones predominantly in the afternoons (PRa: 0.81; 95% CI: 0.68–0.97) (Table 3).

## 4. Discussion

### 4.1. Sleep Quality

About 77.3% of the medical students evaluated reported poor sleep quality, and about half obtained a score for which they possibly required medical attention to treat this problem. A study in 2013 conducted at a medical school in Lambayeque-Peru among medical students from 1st to 6th years, which also used the Pittsburgh sleep quality questionnaire and used 5 points as a cut-off point for poor sleep quality, reported that they presented a prevalence of poor sleep quality in 79.9% of the students evaluated [22], another study in the same year and city, similar to the previous one, evaluated 247 health sciences students, and reported that the 85% of the respondents had a prevalence of poor sleep quality [23].

Although these studies were conducted only in one medical school and with a small number of students, the prevalence of poor sleep quality is similar to what we report, suggesting that medical students in Peru possibly already suffered from poor quality sleep, even before the pandemic. This poor quality of sleep could generate alterations in cognitive processes, memory, and learning [24,25], which would lead to poor academic performance [14,26]. In addition, it has been reported that it can lead to an increased risk of psychological morbidity [27], lowering mood, and finally being related to various mental health problems, cardiovascular diseases, obesity, diabetes, and increased mortality [28]. 

There is controversy about the impact of the COVID-19 pandemic on sleep quality; some authors report that poor sleep quality increased [3,10], probably due to the stress generated by social isolation and changes in habitual behavior. On the other hand, other studies report an improvement in sleep quality [11,29] due to an increase in sleep time due to the fact that classes became online, clinical rotations were not carried out, and the time spent going and coming from the university and hospitals were reduced to zero.

### 4.2. Nomophobia Associated with Poor Sleep Quality

Nomophobia is considered a disorder of contemporary digital and virtual society, and it refers to a pathological fear of being left without a mobile phone. In our research, students with poor sleep quality were associated with higher nomophobia scores. This result is like a study conducted between 2016 and 2017 on 610 medical students from Saudi Arabia [30]. Other investigations on medical students whose instruments differed from ours also associated poor sleep quality with mobile devices [31,32,33]. The similarity of these findings could be because excessive use of mobile devices is a factor in developing sleep disorders [34] due to several mechanisms, such as the bright light emitted by the device [30] and more time looking the cellphone [35]. This may delay the circadian cycle when it occurs at night, which could interfere with sleep quality [32]. It should be noted that these facts may have been amplified in the context of the pandemic.

On the other hand, there is electrophysiological, neuroimmunological, neuroendocrine, and autonomic evidence of arousal and anxiety in people with sleep disorders [36,37,38]. This suggests that anxiety may be a common factor underlying the two conditions (i.e., nomophobia and insomnia). This is consistent with the hyperarousal model of insomnia, and anxiety would also play a prominent role in nomophobia [15]. Therefore, future research should learn more about the mechanisms involved in nomophobia and its possible consequences; this could be developed with multicentric and prospective studies that evaluate not only the characteristics of people with nomophobia but also possible interventions to reduce this health problem.

### 4.3. Anxiety and Depression Associated with Poor Sleep Quality

Students who suffered from symptoms of anxiety or depression had a lower quality of sleep; this result is similar to that of other studies [14,39,40,41]. This association could be because poor sleep quality acts as a symptom, comorbidity, or cause of mental disorders (anxiety or depression) [42] since sleep disturbances can be an early marker of depression or anxiety [43,44].

On the other hand, increased stress related to the COVID-19 pandemic has been associated with longer sleep latency, more fragmented sleep, and nightmares [45,46,47]. These findings are similar to the effects of being in the face of natural disasters or traumas [48]; this could probably be because the COVID-19 pandemic increased stress levels, and this, in turn, caused sleep disorders; moreover, we can’t tell if anxiety directly affected sleep quality or vice versa since we cannot extract a one-way causality with our data. 

### 4.4. Limitations

This study has certain limitations that must be considered when interpreting the results. First, the data analyzed belong to a cross-sectional study, which makes it impossible to assess the direction of the temporality of the associations. Second, all variables of interest were self-reported, with the inherent social desirability bias. However, it was explained to the participants that the survey would be anonymous, which could reduce this risk. Finally, it should be considered that the participants were approached through Facebook and WhatsApp, so the users of these applications are likely overrepresented.

## 5. Conclusions

A high frequency of medical students in Peru had poor sleep quality during the COVID 19 pandemic. In addition, it was shown that there was a significant association between symptoms of anxiety and depression and suffering from Nomophobia. These problems may have been amplified during the pandemic years compared to years before the pandemic. For this reason, it is recommended to carry out educational programs on the importance of sleep and carry out more research on the factors associated with poor sleep quality.

## Figures and Tables

**Table 1:** Characteristics of the study population (*n* = 3139).

Characteristic	*n* (%)
Sex	
Female	1919 (61.1)
Male	1220 (38.9)
Age *	22 (20–24)
18 to 20	1086 (34.6)
21 to 23	1162 (37.0)
24 to more	891 (28.4)
Have a relationship	
Nope	2168 (69.1)
Yes	971 (30.9)
Financing your university	
Public	1544 (49.2)
Private	1595 (50.8)
Year of studies	
1st to 3rd	1609 (51.3)
4th to 6th	1530 (48.7)
Failed a course the previous semester	
Nope	2714 (86.5)
Yes	425 (13.5)
Number of brothers	2 (1–2)
None	264 (8.4)
1 to 2	2122 (67.6)
More than 3	753 (24.0)
Do any sports	
Nope	1660 (52.9)
Yes	1479 (47.1)
The level of education of the parents is higher technical or university	
Mother	2359 (75.2)
Father	2469 (78.7)
How often do you check your cell phone?	
5 min	360 (11.5)
20 min	1841 (58.7)
1 h	598 (19.1)
3 h	340 (10.8)
What part of the day do you use your cell phone?	
All time	411 (13.1)
Mornings	686 (21.9)
Afternoon	934 (29.8)
Night	1108 (35.3)
Anxiety (HSCL-25)	
Score (Range: 10 to 40 points) *	15 (12–20)
Average score ≥ 1.75	1091 (34.8)
Depression (HSCL-25)	
Score (Range: 15 to 60 points) *	25 (19–32)
Average score ≥ 1.75	1343 (42.8)
Nomophobia (NMP-Q)	
Score (Range: 20 to 140 points) *	47 (33–69)
Tertile 1	1047 (33.4)
Tertile 2	1052 (33.5)
Tertile 3	1040 (33.1)
Sleep quality (PSQI)	
Score (Range: 0 to 21 points) *	6 (5–8)
No sleep problem (<5 points)	714 (22.8)
Deserves medical attention (5 to 7 points)	1361 (43.4)
Deserves medical attention and treatment (8 to 14 points)	1042 (33.2)
It is a severe sleep problem (<15 points)	22 (0.7)

* Median (Interquartile Range).

**Table 2:** Score on each PSQI item (*n* = 3139).

Statements	Score (Median ± Interquartile Range)
Dimension 1: Subjective sleep quality	
How would you rate the quality of your sleep during the last four weeks?	1 (1–2)
Dimension 2: Sleep latency	
In the last four weeks, how long did it usually take to fall asleep (fall asleep) at night? (In minutes)	20 (10–30)
In the last four weeks, how long did it usually take to fall asleep (fall asleep) at night? (In hours)	1 (0–2)
Dimension 3: Duration of sleep	
In the last four weeks, how many effective hours have you slept per night? (In hours)	6 (5–7)
Dimension 4: Sleep Efficiency	
In the past four weeks, write the usual time you went to bed	24 (1–23)
In the last four weeks, what time did you usually get out of bed in the morning and have not gone back to sleep?	6 (6–8)
Dimension 5: Sleep disturbances	
In the last four weeks, how many times have you had trouble sleeping because of waking up at night or early in the morning?	1 (0–2)
How many times have you had trouble sleeping for other reasons in the last four weeks?	0 (0–1)
Dimension 6: Use of sleep medications	
During the past four weeks, how often have you taken sleeping medicine (prescribed by the doctor or on your own)?	0 (0–0)
Dimension 7: Diurnal dysfunction	
During the last four weeks, how often have you felt drowsy (or very sleepy) when driving, eating, working, studying, or doing some other activity?	1 (0–2)
Over the past few weeks, how much trouble have you had staying upbeat or excited about doing your tasks or activities?	1 (1–2)

**Table 3:** Poisson regression to determine the factors associated with poor sleep quality in the study population.

Characteristic	Participants with Good Sleep Quality (%)	Participants with Poor Sleep Quality (%)	Crude PR (95% CI)	PR Adjusted (95% CI)
Sex				
Male	861 (70.6)	359 (29.4)	Ref.	Ref.
Female	1214 (63.3)	705 (36.7)	1.24 (1.09–1.40)	1.07 (0.94–1.22)
Age				
18 to 20	695 (64.0)	391 (36.0)	Ref.	Ref.
21 to 23	757 (65.2)	405 (34.9)	0.98 (0.85–1.13)	1.05 (0.89–1.24)
24 or more	623 (69.9)	268 (30.1)	0.85 (0.73–1.00)	1.06 (0.88–1.28)
Have a relationship				
Nope	1415 (65.3)	753 (34.7)	Ref.	-
Yes	660 (68.0)	311 (32.0)	0.92 (0.81–1.05)	-
Financing your university				
Public	1058 (68.5)	486 (31.5)	Ref.	-
Private	1017 (63.8)	578 (36.2)	1.16 (0.99–1.35)	-
Year of studies				
1st to 3rd	1025 (63.7)	584 (36.3)	Ref.	Ref.
4th to 6th	1050 (68.6)	480 (31.4)	0.87 (0.77–0.98)	0.96 (0.83–1.12)
Failed a course the previous semester				
Nope	1825 (67.2)	889 (32.8)	Ref.	Ref.
Yes	250 (58.8)	175 (41.2)	1.26 (1.07–1.48)	1.16 (0.98–1.37)
Number of brothers				
None	161 (61.0)	103 (39.0)	Ref.	-
1 to 2	1405 (66.2)	717 (33.8)	0.87 (0.71–1.07)	-
More than 3	509 (67.6)	244 (32.4)	0.84 (0.66–1.06)	-
Do any sports				
Nope	1032 (62.2)	628 (37.8)	Ref.	Ref.
Yes	1043 (70.5)	436 (29.5)	0.77 (0.69–0.88)	0.94 (0.82–1.06)
His mother’s educational background is higher technical or university				
Nope	552 (70.8)	228 (29.2)	Ref.	-
Yes	1523 (64.6)	836 (35.4)	1.19 (1.03–1.39)	-
His father’s educational background is higher technical or university				
Nope	458 (68.4)	212 (31.6)	Ref.	-
Yes	1617 (65.5)	852 (34.5)	1.07 (0.92–1.25)	-
How often do you check your cell phone?				
5 min	200 (55.6)	160 (44.4)	Ref.	-
20 min	1213 (65.9)	628 (34.1)	0.77 (0.65–0.92)	-
1 h	419 (70.1)	179 (29.9)	0.68 (0.55–0.85)	-
3 h	243 (71.5)	97 (28.5)	0.65 (0.51–0.84)	-
What part of the day do you use your cell phone?				
All time	545 (58.4)	389 (41.7)	Ref.	Ref.
Morning	275 (66.9)	136 (33.1)	0.79 (0.65–0.96)	0.91 (0.75–1.11)
Afternoon	486 (70.9)	200 (29.2)	0.70 (0.59–0.83)	0.81 (0.68–0.97)
Night	769 (69.4)	339 (30.6)	0.74 (0.64–0.86)	0.87 (0.75–1.01)
Anxiety (HSCL-25)				
Average score < 1.75	1589 (77.6)	459 (22.4)	Ref.	Ref.
Average score ≥ 1.75	486 (44.6)	605 (55.5)	2.47 (2.19–2.79)	1.48 (1.27–1.72)
Depression (HSCL-25)				
Average score < 1.75	1454 (81.0)	342 (19.0)	Ref.	Ref.
Average score ≥ 1.75	621 (46.2)	722 (53.8)	2.82 (2.48–3.21)	2.03 (1.72–2.39)
Nomophobia (NMP-Q)				
Tertile 1	801 (76.5)	246 (23.5)	Ref.	Ref.
Tertile 2	694 (66.0)	358 (34.0)	1.45 (1.23–1.70)	1.14 (0.97–1.35)
Tertile 3	580 (55.8)	460 (44.2)	1.87 (1.60–2.19)	1.28 (1.09–1.51)

## Data Availability

Available upon reasonable request.

## References

[1] Martínez-de-Quel Ó., Suárez-Iglesias D., López-Flores M., Pérez C.A. (2021). Physical activity, dietary habits and sleep quality before and during COVID-19 lockdown: A longitudinal study. Appetite.

[2] Ministerio de Salud Gobierno Oficializa Suspensión del Toque de Queda a Nivel Nacional, el Cual Rige Desde el 31 de Enero. https://www.gob.pe/institucion/minsa/noticias/580026-gobierno-oficializa-suspension-del-toque-de-queda-a-nivel-nacional-el-cual-rige-desde-el-31-de-enero.

[3] Marelli S., Castelnuovo A., Somma A., Castronovo V., Mombelli S., Bottoni D., Leitner C., Fossati A., Ferini-Strambi L. (2021). Impact of COVID-19 lockdown on sleep quality in university students and administration staff. J. Neurol..

[4] Huang Y., Zhao N. (2020). Generalized anxiety disorder, depressive symptoms and sleep quality during COVID-19 outbreak in China: A web-based cross-sectional survey. Psychiatry Res..

[5] Miranda-Chavez B., Copaja-Corzo C., Rivarola-Hidalgo M., Taype-Rondan Á. (2022). Fear of Death in Medical Students from a Peruvian University during the COVID-19 Pandemic. Behav. Sci..

[6] Rana W., Mukhtar S., Mukhtar S. (2020). Mental health of medical workers in Pakistan during the pandemic COVID-19 outbreak. Asian J. Psychiatry.

[7] Grandner M.A. (2017). Sleep, Health, and Society. Sleep Med. Clin..

[8] Molla A., Wondie T. (2021). Magnitude of Poor Sleep Hygiene Practice and Associated Factors among Medical Students in Ethiopia: A Cross-Sectional Study. Sleep Disord..

[9] Surani A.A., Zahid S., Surani A., Ali S., Mubeen M., Khan R.H. (2015). Sleep quality among medical students of Karachi, Pakistan. J. Pak. Med. Assoc..

[10] Romero-Blanco C., Rodríguez-Almagro J., Onieva-Zafra M.D., Parra-Fernández M.L., Prado-Laguna M.D.C., Hernández-Martínez A. (2020). Sleep Pattern Changes in Nursing Students during the COVID-19 Lockdown. Int. J. Environ. Res. Public Health.

[11] Luciano F., Cenacchi V., Vegro V., Pavei G. (2021). COVID-19 lockdown: Physical activity, sedentary behaviour and sleep in Italian medicine students. Eur. J. Sport Sci..

[12] Ozcan B., Acimis N.M. (2021). Sleep Quality in Pamukkale University Students and its relationship with smartphone addiction. Pak. J. Med. Sci..

[13] Almojali A.I., Almalki S.A., Alothman A.S., Masuadi E.M., Alaqeel M.K. (2017). The prevalence and association of stress with sleep quality among medical students. J. Epidemiol. Glob. Health.

[14] Rao W.W., Li W., Qi H., Hong L., Chen C., Li C.Y., Ng C.H., Ungvari G.S., Xiang Y.T. (2020). Sleep quality in medical students: A comprehensive meta-analysis of observational studies. Sleep Breath..

[15] Copaja-Corzo C., Aragón-Ayala C.J., Taype-Rondan A., Nomotest-Group (2022). Nomophobia and Its Associated Factors in Peruvian Medical Students. Int. J. Environ. Res. Public Health.

[16] Buysse D.J., Reynolds C.F., Monk T.H., Berman S.R., Kupfer D.J. (1989). The Pittsburgh Sleep Quality Index: A new instrument for psychiatric practice and research. Psychiatry Res..

[17] Royuela A., Macias J.A. (1997). Propiedades clinimetricas de la versión castellana del cuestionario de Pittsburgh. Vigilia-Sueño.

[18] Ministerio de Salud Atención de Víctimas de Violencia, Derechos de Las Personas: Guía Práctica Para Uso en Servicios de Emergencia y Consulta Externa. https://www.gob.pe/institucion/minsa/informes-publicaciones/314088-atencion-de-victimas-de-violencia-derechos-de-las-personas-guia-practica-para-uso-en-servicios-de-emergencia-y-consulta-externa.

[19] Morote R., Hjemdal O., Martinez Uribe P., Corveleyn J. (2017). Psychometric properties of the Resilience Scale for Adults (RSA) and its relationship with life-stress, anxiety and depression in a Hispanic Latin-American community sample. PLoS ONE.

[20] Yildirim C., Correia A.-P. (2015). Exploring the Dimensions of Nomophobia: Development and Validation of a Self-Reported Questionnaire. Comput. Hum. Behav..

[21] León-Mejía A., Calvete E., Patino-Alonso C., Machimbarrena J.M., González-Cabrera J. (2020). Cuestionario de Nomofobia (NMP-Q): Estructura Factorial y Puntos de Corte de La Versión Española. Adicciones.

[22] Del Pielago Meoño A.F., Failoc Rojas V.E., Plasencia Dueñas E.A., Díaz Vélez C. (2013). Calidad de sueño y estilo de aprendizaje en estudiantes de Medicina Humana de la Universidad Nacional Pedro Ruiz Gallo. Acta Médica Peru..

[23] Granados-Carrasco Z., Bartra-Aguinaga A., Bendezú-Barnuevo D., Huamanchumo-Merino J., Hurtado-Noblecilla E., Jiménez-Flores J., León-Jiménez F., Chang-Dávila D. (2013). Calidad del sueño en una facultad de medicina de Lambayeque. An. Fac. Med..

[24] Lim J., Dinges D.F. (2010). A meta-analysis of the impact of short-term sleep deprivation on cognitive variables. Psychol. Bull..

[25] Al-Khani A.M., Sarhandi M.I., Zaghloul M.S., Ewid M., Saquib N. (2019). A cross-sectional survey on sleep quality, mental health, and academic performance among medical students in Saudi Arabia. BMC Res. Notes.

[26] Siddiqui A.F., Al-Musa H., Al-Amri H., Al-Qahtani A., Al-Shahrani M., Al-Qahtani M. (2016). Sleep Patterns and Predictors of Poor Sleep Quality among Medical Students in King Khalid University, Saudi Arabia. Malays. J. Med. Sci..

[27] James B.O., Omoaregba J.O., Igberase O.O. (2011). Prevalence and correlates of poor sleep quality among medical students at a Nigerian university. Ann. Niger. Med..

[28] Yazdi Z., Loukzadeh Z., Moghaddam P., Jalilolghadr S. (2016). Sleep Hygiene Practices and Their Relation to Sleep Quality in Medical Students of Qazvin University of Medical Sciences. J. Caring Sci..

[29] Shrestha D., Adhikari S.P., Rawal N., Budhathoki P., Pokharel S., Adhikari Y., Rokaya P., Raut U. (2021). Sleep quality among undergraduate students of a medical college in Nepal during COVID-19 pandemic: An online survey. F1000Research.

[30] Ibrahim N.K., Baharoon B.S., Banjar W.F., Jar A.A., Ashor R.M., Aman A.A., Al-Ahmadi J.R. (2018). Mobile Phone Addiction and Its Relationship to Sleep Quality and Academic Achievement of Medical Students at King Abdulaziz University, Jeddah, Saudi Arabia. J. Res. Health Sci..

[31] Yang J., Fu X., Liao X., Li Y. (2020). Association of problematic smartphone use with poor sleep quality, depression, and anxiety: A systematic review and meta-analysis. Psychiatry Res..

[32] Nowreen N., Ahad F. (2018). Effect of smartphone usage on quality of sleep in medical students. Natl. J. Physiol. Pharm. Pharmacol..

[33] Jniene A., Errguig L., El Hangouche A.J., Rkain H., Aboudrar S., El Ftouh M., Dakka T. (2019). Perception of Sleep Disturbances due to Bedtime Use of Blue Light-Emitting Devices and Its Impact on Habits and Sleep Quality among Young Medical Students. BioMed Res. Int..

[34] Thomée S., Härenstam A., Hagberg M. (2011). Mobile phone use and stress, sleep disturbances, and symptoms of depression among young adults—A prospective cohort study. BMC Public Health.

[35] Christensen M.A., Bettencourt L., Kaye L., Moturu S.T., Nguyen K.T., Olgin J.E., Pletcher M.J., Marcus G.M. (2016). Direct Measurements of Smartphone Screen-Time: Relationships with Demographics and Sleep. PLoS ONE.

[36] Riemann D., Spiegelhalder K., Feige B., Voderholzer U., Berger M., Perlis M., Nissen C. (2010). The hyperarousal model of insomnia: A review of the concept and its evidence. Sleep Med. Rev..

[37] Kalmbach D.A., Cuamatzi-Castelan A.S., Tonnu C.V., Tran K.M., Anderson J.R., Roth T., Drake C.L. (2018). Hyperarousal and sleep reactivity in insomnia: Current insights. Nat. Sci. Sleep.

[38] Bonnet M.H., Arand D.L. (1997). Hyperarousal and insomnia. Sleep Med. Rev..

[39] Saguem B.N., Nakhli J., Romdhane I., Nasr S.B. (2022). Predictors of sleep quality in medical students during COVID-19 confinement. L’encephale.

[40] Feng G.S., Chen J.W., Yang X.Z. (2005). Study on the status and quality of sleep-related influencing factors in medical college students. Zhonghua Liu Xing Bing Xue Za Zhi.

[41] Perotta B., Arantes-Costa F.M., Enns S.C., Figueiro-Filho E.A., Paro H., Santos I.S., Lorenzi-Filho G., Martins M.A., Tempski P.Z. (2021). Sleepiness, sleep deprivation, quality of life, mental symptoms and perception of academic environment in medical students. BMC Med. Educ..

[42] Ghoreishi A., Aghajani A.H. (2008). Sleep quality in Zanjan university medical students. Tehran Univ. Med. J..

[43] Chang P.P., Ford D.E., Mead L.A., Cooper-Patrick L., Klag M.J. (1997). Insomnia in young men and subsequent depression. The Johns Hopkins Precursors Study. Am. J. Epidemiol..

[44] Ohayon M.M., Caulet M., Priest R.G., Guilleminault C. (1997). DSM-IV and ICSD-90 insomnia symptoms and sleep dissatisfaction. Br. J. Psychiatry.

[45] Gibson R., Shetty H., Carter M., Münch M. (2022). Sleeping in a bubble: Factors affecting sleep during New Zealand’s COVID-19 lockdown. Sleep Adv..

[46] Santini Z.I., Koyanagi A. (2021). Loneliness and its association with depressed mood, anxiety symptoms, and sleep problems in Europe during the COVID-19 pandemic. Acta Neuropsychiatr..

[47] Pesonen A.K., Lipsanen J., Halonen R., Elovainio M., Sandman N., Mäkelä J.M., Antila M., Béchard D., Ollila H.M., Kuula L. (2020). Pandemic Dreams: Network Analysis of Dream Content During the COVID-19 Lockdown. Front. Psychol..

[48] Lavie P. (2001). Sleep disturbances in the wake of traumatic events. N. Engl. J. Med..

